# From learning experience to behavioural intention: examining the effects of animation-based industrial heritage learning among primary school students

**DOI:** 10.3389/fpsyg.2026.1907096

**Published:** 2026-08-24

**Authors:** Xin Bian, Shufang Sun

**Affiliations:** 1 School of Art and Design, Shandong Women’s University, Jinan, China; 2 Wellington School of Architecture, Victoria University of Wellington, Wellington, New Zealand; 3 ZhongQi MaiHe Tianjin Engineering Design and Research Institute Co., Ltd., Tianjin, China

**Keywords:** animation-based learning, behavioural intention, comprehension, engagement, learning experience

## Abstract

Animation-based learning is increasingly used to communicate complex knowledge, yet relatively little is known about how learning experiences are associated with behavioural outcomes. Drawing on multimedia learning theory and interest development theory, this study proposed and tested a perception–process–outcome model in which learning experience is associated with behavioural intention through engagement and perceived comprehension. A quasi-experimental study was conducted with 452 primary school students from Grades 1–6. Students were assigned to either an animation-based learning group (*n* = 226) or a conventional instruction group (*n* = 226). Structural equation modelling and Bootstrap mediation analysis were used to examine the underlying psychological mechanisms. Compared with conventional instruction, animation-based learning produced significantly greater improvements in interest, perceived comprehension, and behavioural intention (all *p* < 0.001). Learning experience was positively associated with engagement (*β* = 0.508) and perceived comprehension (*β* = 0.578), while engagement (β = 0.549) and perceived comprehension (*β* = 0.507) were both positively associated with behavioural intention. Significant indirect effects were observed through engagement (*β* = 0.279) and perceived comprehension (*β* = 0.293). The findings suggest that animation-based learning may support behavioural intention through complementary affective and cognitive pathways. By integrating learning experience, engagement, perceived comprehension, and behavioural intention within a single framework, the study broadens understanding of animation-based learning beyond traditional cognitive outcomes and highlights the importance of combining conceptually clear content with engaging audiovisual design when communicating complex knowledge.

## 1 Introduction

Animation is widely used in education. For learning from animation to be successful, the design and use of animation should consider the perceptual, cognitive, and affective resources of learners. Current theories consider perceptual and cognitive processing to be essential components of learning from animation, particularly in situations where learners must process continuously changing visual information.

According to Lowe (1999), learning from animation requires learners to extract relevant information from a dynamic display at a given moment, retain it in working memory, and integrate it with incoming information over time [see also Ploetzner and Lowe (2012)]. This process involves continuous coordination between perception and cognition, as learners must identify visual events, establish relationships between them, and construct coherent mental representations. As suggested by Luck and Hollingworth (2008) and Schurgin (2018), visuospatial working memory plays a central role in bridging spatial and temporal gaps in perception, making it particularly relevant for learning from animation. Consistent with the Cognitive Theory of Multimedia Learning (Mayer, 2020) and Cognitive Load Theory (Sweller, 2011), effective learning depends on the efficient use of limited cognitive resources. However, research on animation-based learning has largely focused on cognitive constraints, such as task load and processing capacity, while paying less attention to learners’ learning experiences during the learning process, including interest, emotional response, and audiovisual perceptions.

At the same time, learning from animation is not purely a cognitive activity. Learners’ perceptual and affective responses may influence how they attend to and process dynamic visual information. Interest has been shown to support sustained attention and deeper processing (Hidi and Renninger, 2006), while emotional responses can influence engagement and learning outcomes (Pekrun, 2006; Um et al., 2012). In addition, audiovisual experience, as a defining characteristic of animation, may shape both learners’ engagement and their ability to process information. While rich audiovisual presentation can enhance attention and immersion, it may also increase cognitive load if the information is too complex or rapidly presented (Sweller, 2011).

Despite these insights, existing models of animation learning rarely integrate perceptual, affective, and cognitive factors into a unified framework. Most studies focus on immediate learning outcomes, such as comprehension or performance, while paying less attention to how learning experiences influence broader outcomes, such as attitudes or behavioural intentions. As a result, it remains unclear how different dimensions of learners’ experience contribute to meaningful learning processes and outcomes.

This gap is particularly salient in industrial heritage education. Industrial heritage encompasses historical development, technological processes, environmental transformation, and cultural meaning, requiring learners to integrate multiple dimensions of knowledge while developing cultural identity, sustainability awareness, and responsibility towards heritage conservation (Smith, 2006; Ardoin et al., 2020; Bian et al., 2026a). Recent studies have highlighted the potential of digital storytelling and animation to support primary students’ engagement with industrial heritage by facilitating interpretation, meaning-making, and learning in complex educational contexts (Bian et al., 2025a; Bian et al., 2025b). Furthermore, a recent systematic review suggests that although digital storytelling has been increasingly adopted in industrial heritage education, empirical evidence regarding the psychological mechanisms underlying learning and behavioural outcomes remains limited (Bian et al., 2026b). Despite these advances, relatively little research has examined how animation-based learning experiences translate into behavioural intention through learners’ underlying psychological processes. Emerging evidence nevertheless suggests that digital storytelling and multimedia-based learning can promote learners’ environmental awareness, conservation attitudes, and behavioural intentions by combining meaningful narratives with engaging visual representations (Ballantyne et al., 2011; Chen et al., 2025; Guo, 2025). However, these studies have generally focused on overall intervention effectiveness rather than explaining the psychological mechanisms through which animation-based learning influences behavioural intention, particularly among primary school learners.

Furthermore, prior studies have typically examined engagement, comprehension, or emotional responses in isolation. Few studies have integrated these effective and cognitive mechanisms within a single explanatory framework, and even fewer have linked them to behavioural intention outcomes. Distinguishing between affective engagement and cognitive comprehension as parallel pathways is theoretically important because they represent different mechanisms through which experience may shape intention. Emotional investment drives motivation and identity-based action, whereas cognitive understanding may provide the knowledge base for rational commitment (Pekrun, 2006; Sinatra et al., 2015).

It should be acknowledged that models linking engagement and comprehension to learning outcomes are not new to multimedia learning research. The Cognitive-Affective Theory of Learning with Media, for example, proposes that affective and motivational factors influence cognitive engagement with instructional media, thereby contributing to learning (Moreno, 2006). Related multimedia learning research has likewise examined how affective and cognitive processes jointly contribute to immediate outcomes such as comprehension, retention, and learning performance. What remains less well understood is whether these mechanisms also help explain the development of value-based behavioural intentions, particularly when learning concerns socially and culturally meaningful topics rather than neutral instructional content.

Industrial heritage education differs from the content domains more commonly examined in animation-based multimedia learning research, such as biology, physics, or language learning, because its educational aims extend beyond the acquisition of factual knowledge to include the development of cultural understanding, identity, and responsibility toward heritage (Smith, 2006). Accordingly, educational outcomes in this context may include not only cognitive understanding but also learners’ attitudes, values, and intentions to support heritage conservation (Ardoin et al., 2020). It therefore remains unclear whether multimedia learning models developed primarily to explain comprehension-oriented outcomes can be readily generalised to heritage education without empirical investigation (Mayer, 2020). Against this background, the present study examines whether the complementary roles of engagement and comprehension extend beyond immediate cognitive outcomes to value-based behavioural intention. Specifically, it investigates whether these two processes jointly mediate the relationship between animation-based learning experience and support for industrial heritage protection among primary school children, an outcome and learner population that have received relatively limited attention in previous multimedia learning research. By integrating learning experience, engagement, perceived comprehension, and behavioural intention within a single framework, this study seeks to advance understanding of animation-based learning beyond immediate cognitive outcomes and provides a basis for examining broader educational outcomes in heritage education.

To address these gaps, the present study pursues three research questions that together examine both the effectiveness and the underlying mechanisms of animation-based heritage learning:

*RQ1:* Does animation-based learning produce greater gains in interest, perceived comprehension, and behavioural intention toward industrial heritage protection than conventional instruction among primary school students?

*RQ2:* How do learners’ learning experiences, comprising interest, emotional response, and audiovisual experience, influence engagement and perceived comprehension during animation-based learning?

*RQ3:* How do engagement and perceived comprehension contribute to behavioural intention toward industrial heritage protection?

To address these questions, the study adopts a two-stage design. First, a quasi-experimental comparison is conducted to evaluate the effectiveness of animation-based learning relative to conventional instruction (RQ1). Second, structural equation modelling is employed to examine the psychological mechanisms underlying the intervention effect (RQ2 and RQ3). Building on a perception–process–outcome framework, the study proposes and tests a dual-pathway model in which learning experience influences behavioural intention through the parallel mediating roles of engagement and comprehension.

## 2 Theoretical and empirical background

### 2.1 Learning from animation in complex knowledge contexts

Learning from animation has been widely applied in educational contexts, particularly when learners need to understand complex and dynamic processes. Prior research suggests that animation can make abstract, invisible, or temporally changing information more explicit and visually accessible, thereby supporting conceptual understanding. For example, Thatcher (2006) showed that animation improved students’ understanding of biological processes compared with traditional text-based materials, while Roohani et al. (2015) found that animated visualisations in multimedia environments enhanced reading comprehension when combined with instructional guidance. Similar benefits have been documented in medical and health science education, where multimedia and animation have been shown to support understanding of complex anatomical and embryological content (Abas et al., 2022; Elbeshbeishy et al., 2025). Comparable improvements in educational effectiveness have also been reported in technology-enhanced instructional environments. For example, Hessen et al. (2026) found that AI-supported blended learning significantly improved students’ academic achievement compared with conventional instruction. These studies indicate that animation can help learners process knowledge that is difficult to explain through static representations alone.

This benefit is particularly relevant when learning content is conceptually demanding. For example, Lee et al. (2019) noted that students often have difficulty understanding complex relationships in biochemical pathways, while Tsehay Mengistie (2025) reported that animation-supported instruction improved conceptual understanding of complex geographical processes. Yang et al. (2026) further demonstrated that three-dimensional animation addressing the most challenging points in oral histology and embryology significantly improved student learning performance. Similarly, Leshko et al. (2025) found that integrating historical and animated elements into physics instruction enhanced students’ scientific competencies. These findings suggest that animation is especially valuable when the subject matter involves dynamic change, multiple relationships, and abstract structures.

In the present study, this challenge is reflected in industrial heritage education. Industrial heritage requires learners to understand interconnected historical, technological, environmental, spatial, and cultural dimensions, all of which can be difficult for primary school students to grasp through conventional explanation alone. Unlike many school subjects that focus primarily on factual knowledge, industrial heritage requires learners to connect historical events, technological systems, places, and social values across time. Such complexity places considerable demands on young learners’ cognitive resources.

At the same time, highly interactive multimedia formats may impose additional processing demands. Ross et al. (2016) found that excessive interactivity in digital storybooks negatively affected children’s comprehension, even though it increased emotional engagement. Bakan et al. (2025) similarly observed that the visual design of virtual characters influences learners’ attitudes and reading comprehension, suggesting that perceptual features of animated environments can either support or impede understanding. This suggests that, for young learners dealing with conceptually complex content, overly demanding formats may distract attention from essential information rather than support understanding.

For this reason, animation was selected as a structured and guided mode of representation. Compared with highly interactive multimedia systems, animation can present key processes and relationships in a more controlled manner, allowing learners to focus on essential information while maintaining interest and attention. Animation also provides opportunities to combine narrative structure, visual representation, and emotional storytelling, thereby supporting both cognitive processing and learner engagement. Cognitive demands are therefore treated as an important design consideration in this study, whereas the primary analytical focus is on how learners’ experiences during animation-based learning relate to subsequent learning processes and behavioural outcomes.

### 2.2 Learning experience, engagement, and comprehension

Learning from animation is influenced not only by the objective characteristics of instructional materials but also by learners’ subjective experiences during the learning process. Previous research suggests that interest, emotional response, and audiovisual experience play important roles in shaping how learners attend to, interpret, and retain information presented in multimedia environments (Hidi and Renninger, 2006; Mayer, 2020; Pekrun, 2006). Collectively, these factors can be understood as dimensions of learning experience, reflecting learners’ affective and perceptual responses to instructional content.

Among these dimensions, interest is often regarded as a key driver of learning. Interest encourages learners to allocate attention and cognitive resources to learning tasks, thereby promoting deeper information processing and sustained motivation (Hidi and Renninger, 2006). Emotional responses also influence learning by shaping learners’ willingness to engage with content and their perceptions of its personal relevance (Pekrun, 2006). In addition, audiovisual experience, as a defining characteristic of animation, may affect learners’ perceptions of content quality, enjoyment, and accessibility. Well-designed audiovisual elements can facilitate information processing and maintain attention, whereas poorly designed elements may create unnecessary cognitive load (Mayer, 2020).

Learning experience is closely associated with engagement. Although these concepts are related, they represent different aspects of learning. Interest, emotional response, and audiovisual experience reflect learners’ perceptions and reactions to instructional materials, whereas engagement refers to the extent to which learners sustain attention, effort, and involvement throughout the learning process. Prior studies have shown that positive learning experiences can enhance engagement by encouraging learners to invest greater cognitive and emotional resources in learning activities (Fredricks et al., 2004; Sinatra et al., 2015).

Learning experience may also influence comprehension. According to the Cognitive Theory of Multimedia Learning (Mayer, 2020), meaningful learning occurs when learners actively select, organise, and integrate information from multiple channels. Positive emotional and perceptual experiences may facilitate this process by supporting attention, reducing resistance to learning, and increasing willingness to process information. Consequently, learners who find instructional materials interesting, emotionally meaningful, and audiovisually engaging may be more likely to achieve deeper understanding of complex concepts.

Both engagement and comprehension have been associated with broader attitudinal and behavioural outcomes. Engagement can foster personal involvement and motivational commitment, whereas comprehension provides the knowledge foundation necessary for informed judgement and behavioural support. In heritage education contexts, learners who are both emotionally engaged and cognitively informed may be more likely to develop supportive attitudes and intentions toward heritage preservation. However, these relationships should not be assumed to operate uniformly across all multimedia learning contexts. The cognitive demands imposed by instructional design and the complexity of the learning content may influence whether positive learning experiences are translated into meaningful understanding and subsequent behavioural outcomes (Mayer, 2020; Sweller, 2011). This consideration is particularly relevant in industrial heritage education, where learners must integrate historical, technological, environmental, and cultural information simultaneously. This suggests that engagement and comprehension may function as complementary pathways linking learning experience to behavioural intention, with engagement representing an affective pathway and comprehension representing a cognitive pathway. The extent to which these pathways operate in animation-based industrial heritage learning therefore requires empirical investigation.

### 2.3 Empirical research

To identify empirical studies examining animation-based learning and its relationships with engagement, comprehension, emotional response, and related learning outcomes, a systematic search was conducted in March 2026 using the Scopus database. The search expression combined terms related to animation, learning, engagement, emotional response, interest, comprehension, and student populations: TITLE-ABS-KEY (animation) AND TITLE-ABS-KEY (learning OR “multimedia learning”) AND TITLE-ABS-KEY (engagement OR “emotional response” OR interest) AND TITLE-ABS-KEY (comprehension) AND TITLE-ABS-KEY (student* OR children). Further limitations were year of publication (2006–2026), document type (research article), and language (English).

The search initially yielded 74 articles. The following criteria were used to identify studies relevant to the present research:

- Animation had to be the primary instructional medium rather than a minor component of a broader multimedia environment.
- Participants had to be school-age learners or students.
- The study had to report empirical findings based on quantitative, qualitative, or mixed-methods data.
- Learning outcomes had to include at least one of the following: engagement, emotional response, learning experience, comprehension, or related educational outcomes.

The titles and abstracts of the 74 articles were reviewed. Articles that violated one or more of the selection criteria were excluded. This process resulted in 25 studies for detailed review and analysis. The reviewed studies revealed three major research trends.

First, a substantial proportion of studies focused on the role of animation in facilitating comprehension and learning performance. Thatcher (2006) reported that animation improved students’ understanding of biological processes compared with traditional text-based materials. Roohani et al. (2015) similarly found that animated visualisations enhanced reading comprehension when combined with instructional guidance. In geography education, Tsehay Mengistie (2025) showed that animation-supported instruction improved conceptual understanding of complex geographical processes, while Yang et al. (2026) demonstrated that three-dimensional animation enhanced learning performance in oral histology and embryology. Similar benefits have also been reported in science and technology education, where animation helped learners understand complex concepts involving dynamic relationships and abstract structures (Mahadevaswamy et al., 2025; Subramanian and Inukurthi, 2026; Turkay, 2022). Collectively, these findings suggest that animation is particularly effective for communicating complex and dynamically changing knowledge.

Second, many studies examined the influence of animation on learners’ affective experiences. Sandaran and Kia (2013) found that animation increased learners’ interest and motivation, while Anggraeni et al. (2019) reported improvements in attention and enjoyment. Hoyt et al. (2025) further observed that animation-based learning environments enhanced learner engagement and emotional involvement. Similarly, Marzouk and Ibrahim (2026) and Lu et al. (2024) reported positive effects on learning experience and emotional response. Other studies likewise highlighted the potential of animation to increase attention, enjoyment, and motivational engagement (Ashfaq and Ansari, 2020; Liu et al., 2024; Wagino et al., 2025). Together, these findings indicate that animation may influence not only cognitive outcomes but also learners’ emotional and motivational responses.

Although both cognitive and affective benefits have been widely reported, relatively few studies have examined how these dimensions interact with one another. Third, only a limited number of studies simultaneously investigated relationships between cognitive and affective outcomes. Kim et al. (2007) reported that animation increased learners’ interest and motivation without significantly improving comprehension. Similarly, Ross et al. (2016) found that highly interactive multimedia environments enhanced engagement but reduced comprehension. These findings suggest that positive learning experiences do not necessarily translate directly into improved understanding. As a result, the relationships between experiential, affective, and cognitive variables remain insufficiently understood.

Overall, the reviewed literature suggests that animation can support both comprehension and positive learning experiences. However, most studies have treated comprehension, engagement, motivation, and emotional response as separate constructs, with relatively few investigating how these variables operate together within a unified explanatory framework. Furthermore, behavioural outcomes have received relatively less attention than cognitive outcomes in the animation-based learning literature. Some studies using digital storytelling, interactive multimedia, and other narrative-based formats have suggested possible links with learners’ pro-environmental attitudes and behavioural intentions (Chen et al., 2025; Bian et al., 2026b). However, evidence directly examining behavioural intention in animation-based learning remains comparatively limited, particularly regarding the psychological processes through which learning experiences may relate to subsequent intentions.

This limitation is particularly evident in heritage education, where effective learning involves not only understanding historical and cultural knowledge but also developing supportive attitudes toward heritage conservation. Despite growing interest in digital heritage communication, little empirical research has examined how animation-based learning experiences are transformed into behavioural intentions through both affective and cognitive processes. Research focusing on primary school learners is especially limited, even though this stage is critical for developing heritage awareness, value formation, and future conservation attitudes.

To address these gaps, the present study proposes a perception–process–outcome framework in which learning experience, comprising interest, emotional response, and audiovisual experience, influences supportive intention toward industrial heritage protection through two parallel pathways: engagement and comprehension. By combining a quasi-experimental design with structural equation modeling, the study seeks to explain not only whether animation-based learning is effective, but also how learning experiences contribute to behavioural intention in heritage education contexts.

## 3 Method and materials

### 3.1 Design and hypotheses

To examine both the effectiveness and potential mechanisms of animation-based industrial heritage learning, this study adopted a two-stage quantitative design combining a quasi-experimental comparison with structural equation modeling (SEM). In the first stage, a pre-test–post-test quasi-experimental design involving an experimental group and a control group was employed to evaluate the effectiveness of the animation intervention. Interest, perceived comprehension, and supportive intention toward industrial heritage protection were measured before and after the intervention. In the second stage, SEM was conducted to examine the relationships among learning experience, engagement, perceived comprehension, and supportive intention toward industrial heritage protection. Learning experience was conceptualised as a higher-order construct consisting of interest, emotional response, and audiovisual experience. Although previous research generally supports positive associations among learning experience, engagement, perceived comprehension, and educational outcomes, findings from animation-based learning also suggest that these relationships may vary according to instructional design and the complexity of the learning content (Kim et al., 2007; Ross et al., 2016). Accordingly, the proposed relationships require empirical examination in the context of animation-based industrial heritage learning. Five hypotheses were tested.

*H1:* Positive learning experiences may encourage greater involvement in learning activities. Accordingly, learning experience is predicted to be positively associated with engagement.

*H2:* Positive learning experiences may facilitate information processing and understanding. Therefore, learning experience is predicted to be positively associated with perceived comprehension.

*H3:* Previous studies have reported positive associations between learner engagement and educational outcomes (Fredricks et al., 2004; Sinatra et al., 2015). Accordingly, engagement is predicted to be positively associated with supportive intention toward industrial heritage protection.

*H4:* Perceived comprehension may contribute to learners’ understanding of the significance and value of industrial heritage. Therefore, perceived comprehension is predicted to be positively associated with supportive intention toward industrial heritage protection.

*H5:* Learning experience may influence supportive intention through both affective and cognitive processes. Consequently, engagement and perceived comprehension are predicted to mediate the relationship between learning experience and supportive intention toward industrial heritage protection.

### 3.2 Participants

A total of 470 questionnaires were distributed, and all were returned. After excluding invalid responses, 452 valid questionnaires were retained, yielding an effective response rate of 96.17%. Participants were primary school students from Grade 1 to Grade 6, including 72 Grade 1 students (15.93%), 68 Grade 2 students (15.04%), 88 Grade 3 students (19.47%), 68 Grade 4 students (15.04%), 73 Grade 5 students (16.15%), and 83 Grade 6 students (18.36%). The valid sample was equally divided into an experimental group (*n* = 226) and a control group (*n* = 226), with no significant difference in grade composition between groups (*p* = 0.935). Ethical approval was obtained from the Human Ethics Committee of Victoria University of Wellington (Approval No. 0000030449), and written informed consent was obtained from parents or legal guardians before participation. For the SEM analysis, only post-test data from the experimental group were used.

### 3.3 Materials

#### 3.3.1 Educational animation

**Figure 1:**
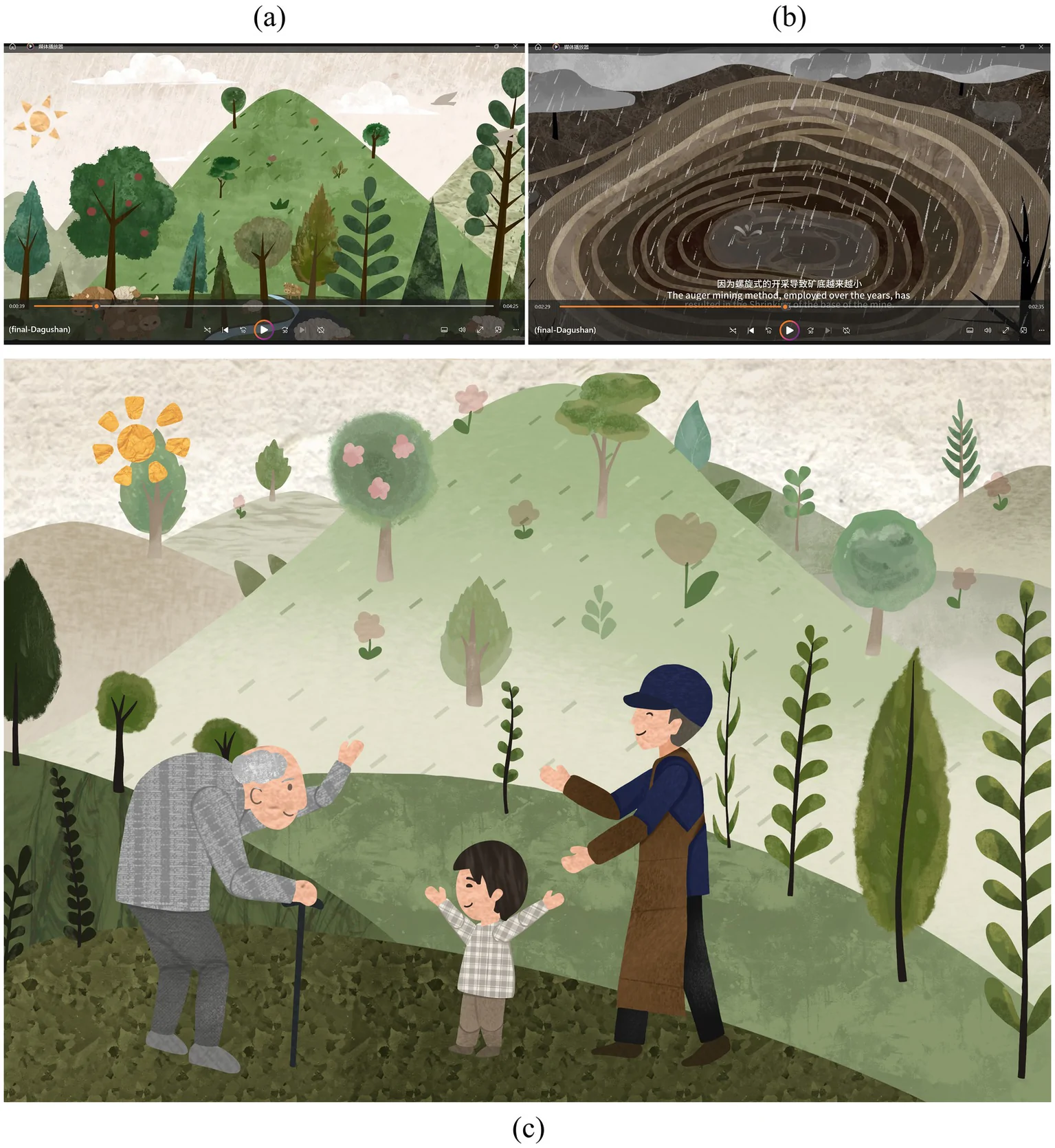
Representative scenes from the educational animation Centennial Mine: Dagushan: **(a)** Opening scene depicting the natural landscape of Dagushan, **(b)** Mining scene illustrating open-pit extraction activities, and **(c)** The three-generation miner family serving as the central narrative framework.

The instructional material was an educational animation entitled *Centennial Mine: Dagushan*, developed specifically for industrial heritage education. The animation presents the historical development, industrial transformation, and environmental changes of the Dagushan mining site in a format suitable for primary school students. It adopts a narrative structure centred on a three-generation miner family, through which more than a century of industrial development is conveyed. The animation has a total duration of 5 min 2 s and employs a hand-drawn visual style, structured scene transitions, and a retro colour palette (Figure 1). Voice-over narration, subtitles, background music, and sound effects were incorporated to support understanding and enhance the viewing experience. Through visual storytelling and chronological narration, the animation presents complex industrial heritage concepts in a form accessible to young learners. Consistent with multimedia learning principles (Mayer, 2020), the animation adopted a relatively simple visual design and linear narrative structure, avoiding highly interactive or information-dense elements that might impose unnecessary processing demands on primary school learners. This design aimed to support learners’ processing of key historical, technological, and environmental information while maintaining engagement with the content.

#### 3.3.2 Conventional instructional materials

Students in the control group received conventional learning materials. The materials included teacher-guided explanations and written resources presenting the historical background, industrial development, and environmental transformation of the Dagushan mining site. The instructional content was equivalent to that presented in the animation but was delivered through conventional classroom teaching without animation or multimedia elements. The duration of the learning activity was matched to that of the animation intervention, and both groups completed the learning activities under comparable classroom conditions.

### 3.4 Procedure

The intervention was conducted during regular school hours. Participants were randomly selected from the eligible student population within each grade, and the intervention was implemented at the individual student level rather than by assigning intact classrooms. First, all participants completed a pre-test questionnaire assessing interest, perceived comprehension, and supportive intention toward industrial heritage protection. Students in the experimental group then watched *Centennial Mine: Dagushan*, whereas those in the control group received conventional learning materials covering the same industrial heritage content. Apart from the instructional format, the learning content, duration, classroom setting, and assessment procedures were kept consistent across the two groups. Following the learning activity, all participants completed a post-test questionnaire. Students in the experimental group additionally completed measures of emotional response, audiovisual experience, and engagement, which were subsequently used in the SEM analysis. The entire procedure was completed within approximately 20–25 min.

### 3.5 Measures

All questionnaire items employed a five-point Likert scale ranging from 1 (strongly disagree) to 5 (strongly agree). The pre-test and post-test questionnaires administered to both groups assessed three core constructs: interest, perceived comprehension, and behavioural intention toward industrial heritage protection. The post-test questionnaire administered to the experimental group additionally assessed emotional response, audiovisual experience, and engagement. Together with interest, these variables served as indicators of the higher-order learning experience construct used in the SEM analysis. All measurement items were adapted from validated instruments in multimedia learning, educational psychology, and heritage education research. Perceived comprehension was assessed as students’ self-reported understanding of the learning content rather than objective knowledge performance. The complete questionnaire items are provided in Supplementary Table S5.

### 3.6 Data analysis

Data analysis was conducted in two stages using SPSS 27.0 and AMOS. First, independent-samples *t*-tests were used to compare the experimental and control groups and paired-samples *t*-tests were conducted to examine within-group changes between pre-test and post-test measurements. In addition to *p*-values, Cohen’s *d* was reported as the standardised mean difference, partial eta squared (η^2^p) was reported as an additional measure of effect size, and 95% confidence intervals were calculated for the unstandardised mean differences. Only complete questionnaires were retained for analysis; therefore, no missing data treatment was required. Data normality was examined using skewness and kurtosis, no influential multivariate outliers were identified based on Mahalanobis distance, and multicollinearity was assessed using the full collinearity test (Kock, 2015), with variance inflation factor (VIF) values ranging from 1.233 to 2.173. Second, before evaluating the measurement and structural models, potential common method bias was assessed using Harman’s single-factor test and by comparing the hypothesised measurement model with an alternative single-factor model. The reliability and validity of the measurement model were then evaluated using Cronbach’s alpha, composite reliability (CR), average variance extracted (AVE), and confirmatory factor analysis (CFA). Structural equation modelling (SEM) was subsequently performed in AMOS to test the hypothesised relationships, and indirect effects were evaluated using bootstrap resampling with 5,000 samples.

## 4 Results

### 4.1 Educational effectiveness of the animation intervention

#### 4.1.1 Baseline equivalence between experimental and control groups

Before the intervention, baseline equivalence between the experimental and control groups was examined. Grade composition did not differ significantly between the two groups (*p* = 0.935). As shown in Table 1, independent-samples t-tests indicated no significant differences in interest, comprehension, or behavioural intention toward industrial heritage protection at pre-test (all *p* > 0.05), suggesting satisfactory comparability between groups prior to the intervention.

**Table 1:** Pre-test comparison between the experimental and control groups.

Variable	Experimental (*N* = 226)	Control (*N* = 226)	Mean difference (95% CI)	Cohen’s *d*	η^2^*p*	*t*	*p*
Interest	2.839 ± 0.404	2.798 ± 0.454	−0.041 (−0.120, 0.038)	−0.096	0.002	−1.021	0.308
Comprehension	2.878 ± 0.397	2.816 ± 0.373	−0.062 (−0.133, 0.009)	−0.161	0.006	−1.710	0.088
Enthusiasm for Heritage Protection	2.814 ± 0.426	2.794 ± 0.436	−0.020 (−0.097, 0.057)	−0.048	0.001	−0.509	0.611

Values are presented as mean ± standard deviation (M ± SD). Independent-samples *t*-tests were conducted to compare pre-test scores between the experimental and control groups. Mean differences and Cohen’s *d* were calculated as control minus experimental and are presented with 95% confidence intervals (CI). Partial eta squared (η^2^p) is reported as an additional effect-size measure.

#### 4.1.2 Post-test differences between the experimental and control groups

Following the intervention, significant between-group differences emerged across all three outcome variables (Table 2; all *p* < 0.001). Students in the experimental group reported higher levels of interest, comprehension, and behavioural intention toward industrial heritage protection than those in the control group.

**Table 2:** Post-test comparison between the experimental and control groups (Experimental: *N* = 226; Control: *N* = 226).

Variable	Experimental (*N* = 226)	Control (*N* = 226)	Mean difference (95% CI)	Cohen’s *d*	η^2^*p*	*t*	*p*
Interest	3.923 ± 0.430	3.158 ± 0.360	−0.765 (−0.838, −0.692)	−1.931	0.484	−20.528	< 0.001
Comprehension	3.886 ± 0.380	3.180 ± 0.429	−0.706 (−0.781, −0.631)	−1.744	0.433	−18.535	< 0.001
Enthusiasm for heritage protection	3.882 ± 0.403	3.174 ± 0.421	−0.708 (−0.784, −0.632)	−1.718	0.426	−18.258	< 0.001

Values are presented as mean ± standard deviation (M ± SD). Independent-samples *t*-tests were conducted to compare post-test scores between the experimental and control groups. Mean differences and Cohen’s *d* were calculated as control minus experimental and are presented with 95% confidence intervals (CI). Partial eta squared (η^2^p) is reported as an additional effect-size measure.

#### 4.1.3 Within-group changes from pre-test to post-test

Paired-samples t-tests were conducted to examine changes within each group. As shown in Table 3, significant increases were observed in interest, comprehension, and behavioural intention in the experimental group from pre-test to post-test (all *p* < 0.001). As shown in Table 4, the control group also improved significantly from pre-test to post-test across all three variables (all *p* < 0.001). Although significant improvements were observed in both groups, the magnitude of change was substantially larger in the experimental group. For example, interest increased by 1.084 points in the experimental group compared with 0.360 points in the control group. Similar patterns were observed for comprehension (1.009 vs. 0.364) and behavioural intention (1.068 vs. 0.381), indicating stronger learning gains following the animation intervention.

**Table 3:** Pre-test and post-test comparison in the experimental group (*N* = 226).

Variable	Pre-test	Post-test	Mean difference (95% CI)	Cohen’s *dz*	η^2^*p*	*t*	*p*
Interest	2.839 ± 0.404	3.923 ± 0.430	−1.084 (−1.158, −1.010)	−1.917	0.787	−28.815	<0.001
Comprehension	2.878 ± 0.397	3.886 ± 0.380	−1.009 (−1.075, −0.943)	−2.002	0.801	−30.097	<0.001
Enthusiasm for heritage protection	2.814 ± 0.426	3.882 ± 0.403	−1.068 (−1.141, −0.995)	−1.908	0.785	−28.679	<0.001

Values are presented as mean ± standard deviation (M ± SD). Mean differences and Cohen’s *dz* were calculated as pre-test minus post-test and are presented with 95% confidence intervals (CI). Paired-samples *t*-tests were conducted within the experimental group. Partial eta squared (η^2^*p*) is reported as an additional effect-size measure.

**Table 4:** Pre-test and post-test comparison in the control group (*N* = 226).

Variable	Pre-test	Post-test	Mean difference (95% CI)	Cohen’s *dz*	η^2^*p*	*t*	*p*
Interest	2.798 ± 0.454	3.158 ± 0.360	−0.360 (−0.432, −0.288)	−0.652	0.299	−9.800	<0.001
Comprehension	2.816 ± 0.373	3.180 ± 0.429	−0.364 (−0.439, −0.289)	−0.640	0.291	−9.617	<0.001
Enthusiasm for heritage protection	2.794 ± 0.436	3.174 ± 0.421	−0.381 (−0.458, −0.304)	−0.651	0.298	−9.783	<0.001

Values are presented as mean ± standard deviation (M ± SD). Mean differences and Cohen’s *dz* were calculated as pre-test minus post-test and are presented with 95% confidence intervals (CI). Paired-samples *t*-tests were conducted within the control group. Partial eta squared (η^2^*p*) is reported as an additional effect-size measure.

#### 4.1.4 Summary of quasi-experimental findings

Overall, the experimental and control groups were comparable before the intervention. After the intervention, the experimental group showed significantly higher post-test scores and larger pre-post gains than the control group across all three outcome variables. These findings provide evidence for the educational effectiveness of the animation intervention. Having established the effectiveness of the intervention, the following section examines the psychological mechanisms through which animation-based learning contributes to these outcomes.

### 4.2 Mechanisms of animation-based learning

Before evaluating the measurement model, potential common method bias was first examined. Harman’s single-factor test extracted five factors with eigenvalues greater than 1, and the first factor accounted for 34.08% of the total variance, below the recommended threshold of 50% (Podsakoff et al., 2003). Furthermore, the hypothesised six-factor measurement model demonstrated a substantially better fit than an alternative single-factor model (Supplementary Table S4), indicating that a single common factor did not adequately account for the observed covariance structure. Together, these findings suggest that common method bias is unlikely to have materially influenced the results. The overall scale demonstrated good internal consistency (Cronbach’s *α* = 0.883). Cronbach’s alpha coefficients for the six first-order constructs ranged from 0.824 to 0.847, indicating satisfactory reliability. The Kaiser–Meyer–Olkin value was 0.860, and Bartlett’s test of sphericity was significant (*χ*^2^ = 1927.309, df = 153, *p* < 0.001), confirming the suitability of the data for factor analysis. Item-level reliability statistics, including corrected item–total correlations (CITC) and Cronbach’s alpha if an item was deleted, are presented in Table 5.

**Table 5:** CITC values and Cronbach’s alpha by dimension.

Dimension	Item	CITC	α if item deleted	Cronbach’s *α*
Interest	INT1	0.689	0.765	0.830
	INT2	0.685	0.769	
	INT3	0.692	0.762	
Emotional response	ER1	0.702	0.751	0.830
	ER2	0.656	0.796	
	ER3	0.708	0.745	
Visual and auditory experience	VAE1	0.726	0.745	0.836
	VAE2	0.663	0.805	
	VAE3	0.704	0.766	
Engagement	ENG1	0.637	0.799	0.824
	ENG2	0.713	0.725	
	ENG3	0.691	0.747	
Comprehension	COM1	0.669	0.779	0.828
	COM2	0.699	0.749	
	COM3	0.689	0.759	
Behavioural intention	BI1	0.709	0.792	0.847
	BI2	0.730	0.773	
	BI3	0.706	0.796	

CITC, Corrected Item–Total Correlation. *α* if item deleted indicates the Cronbach’s alpha coefficient after removing the corresponding item. All items demonstrated acceptable item–total correlations (CITC >0.50), and the deletion of any individual item did not improve scale reliability, supporting the retention of all items.

Confirmatory factor analysis (CFA) was subsequently conducted to evaluate the measurement model. Standardised factor loadings ranged from 0.713 to 0.840, indicating satisfactory indicator reliability. Composite reliability (CR) values ranged from 0.826 to 0.847, exceeding the recommended threshold of 0.70, while average variance extracted (AVE) values ranged from 0.614 to 0.649, exceeding the recommended threshold of 0.50 and supporting satisfactory convergent validity. Detailed measurement model results, including standardised factor loadings, composite reliability (CR), and average variance extracted (AVE), are provided in Supplementary Table S1.

Discriminant validity was assessed using the heterotrait–monotrait ratio (HTMT). HTMT values ranged from 0.244 to 0.695, remaining below the recommended threshold of 0.85 and supporting adequate discriminant validity among the latent constructs. Further details of the discriminant validity assessment and model diagnostics, including the complete HTMT matrix and modification indices, are presented in Supplementary Tables S2, S3, respectively. Collectively, these findings support the reliability and validity of the measurement model and provide an appropriate basis for subsequent structural equation modelling (see Table 6).

**Table 6:** KMO and Bartlett’s test of sphericity.

Statistic	Value
KMO measure of sampling adequacy	0.860
Bartlett’s test–Approx. *χ*^2^	1927.309
*df*	153
Sig.	< 0.001

KMO, Kaiser–Meyer–Olkin measure of sampling adequacy; df, degrees of freedom.

### 4.3 Structural equation modeling results

Following confirmation of measurement model fit, the structural model was estimated using post-test data from the experimental group (*n* = 226). The SEM specified learning experience as a second-order latent variable with three first-order indicators (interest, emotional response, and audiovisual experience), with engagement and comprehension as parallel mediators and behavioural intention as the outcome variable.

#### 4.3.1 Structural model fit

Model fit indices are presented in Table 7. The structural model demonstrated a good overall fit to the data (*χ*^2^ = 110.928, df = 126, *χ*^2^/df = 0.880, RMSEA = 0.000, NFI = 0.944, CFI = 1.000, IFI = 1.008, TLI = 1.010, GFI = 0.950, AGFI = 0.932; *p* = 0.828). All fit indices met or exceeded commonly recommended thresholds, indicating good agreement between the proposed model and the observed covariance matrix. Because the *χ*^2^/df value was below 1, the RMSEA value of 0.000 and the IFI and TLI values slightly above 1.00 are mathematically consistent with the computation of these fit indices when *χ*^2^ is smaller than the model degrees of freedom.

**Table 7:** Structural model fit indices.

Index	Criterion	Value
*χ* ^2^	—	110.928
df	—	126.000
*χ*^2^/df	<3.0	0.880
RMSEA	<0.08	0.000
NFI	>0.90	0.944
CFI	>0.90	1.000
IFI	>0.90	1.008
RFI	>0.90	0.932
TLI	>0.90	1.010
GFI	>0.90	0.950
AGFI	>0.90	0.932

RMSEA, Root Mean Square Error of Approximation; NFI, Normed Fit Index; CFI, Comparative Fit Index; IFI, Incremental Fit Index; RFI, Relative Fit Index; TLI, Tucker–Lewis Index; GFI, Goodness-of-Fit Index; AGFI, Adjusted Goodness-of-Fit Index.

#### 4.3.2 Direct effects and hypothesis testing

Standardised path coefficients, standard errors, and critical ratios for the structural model are presented in Table 8. All four hypothesised direct paths were positive and statistically significant (all *p* < 0.001). As shown in Table 8, learning experience was positively associated with engagement and comprehension, which were in turn positively associated with behavioural intention toward industrial heritage protection. Among the four direct effects, the path from learning experience to comprehension showed the largest standardised coefficient (*β* = 0.578). Accordingly, H1, H2, H3, and H4 were supported.

**Table 8:** Standardised path coefficients for direct effects.

Hypothesis	Path	Std. *β*	S.E.	C. R. (*t*)	*p*
H1	Learning experience → Engagement	0.508	0.055	3.805	***
H2	Learning experience → Comprehension	0.578	0.064	3.998	***
H3	Engagement → Behavioural intention	0.549	0.080	7.589	***
H4	Comprehension → Behavioural intention	0.507	0.070	7.421	***

Std. β, standardised path coefficient; S.E., standard error; C. R., critical ratio. *** indicates *p* < 0.001.

#### 4.3.3 Mediation analysis

Bootstrap resampling (*n* = 5,000) was used to test the indirect effects of learning experience on behavioural intention through engagement and comprehension. As shown in Table 9, both indirect paths were significant (*p* < 0.001). The indirect effect through engagement was 0.279, while the indirect effect through comprehension was 0.293. The total indirect effect was 0.572. These results support H5.

**Table 9:** Bootstrap mediation results (*N* = 5,000 resamples).

Mediation path	Std. indirect effect	Significance
LE → engagement → behavioural intention	0.279	***
LE → comprehension → behavioural intention	0.293	***
Total indirect effect	0.572	***

LE, Learning Experience. Indirect effects were estimated using 5,000 bootstrap resamples. *** indicates *p* < 0.001.

#### 4.3.4 Summary of structural model findings

The SEM results supported all five proposed hypotheses. Learning experience was positively associated with behavioural intention through two parallel mediating pathways: engagement and comprehension. Bootstrap analyses confirmed the significance of both indirect effects, which were similar in magnitude. These findings support the proposed dual-pathway mechanism, suggesting that stronger learning experiences are associated with greater behavioural intention toward industrial heritage protection through higher levels of engagement and comprehension.

## 5 Discussion

### 5.1 Learning experience as a driver of engagement and comprehension

The quasi-experimental results showed that students who participated in animation-based learning demonstrated greater improvements in interest, perceived comprehension, and behavioural intention than those receiving conventional instruction. This finding suggests that animation may provide advantages over conventional learning materials when communicating complex industrial heritage content to primary school students.

The present study found that learning experience significantly predicted both engagement (*β* = 0.508) and perceived comprehension (*β* = 0.578). This finding indicates that students’ responses to animation-based learning were not limited to immediate enjoyment or preference but were also associated with core learning processes. In other words, when students experienced the animation as interesting, emotionally meaningful, and audiovisually engaging, they were more likely to remain engaged and to report stronger comprehension of the learning content.

This result extends previous research on animation-based learning. Prior studies have shown that animation can support attention, motivation, and understanding in complex learning contexts (Mayer, 2020; Roohani et al., 2015; Schnotz and Rasch, 2005; Thatcher, 2006). Building on this work, the present study suggests that the effectiveness of animation is related not only to the objective features of the instructional material, but also to learners’ subjective experience during the learning process.

The stronger path from learning experience to comprehension (*β* = 0.578) is particularly meaningful from a multimedia learning perspective. According to the Cognitive Theory of Multimedia Learning, learners construct understanding by selecting, organising, and integrating verbal and visual information (Mayer, 2020). Similarly, Schnotz and Rasch (2005) argued that animation supports learning when it facilitates meaningful cognitive processing rather than merely attracting attention. In this study, the Dagushan animation combined narration, visual storytelling, and historical content within a coherent narrative sequence. Unlike conventional text-based learning materials, the animation presented industrial development, mining activities, and environmental transformation through the experiences of a three-generation miner family. This narrative approach may have helped students connect abstract industrial processes with human experiences, thereby supporting both engagement and perceived comprehension.

The relationship between learning experience and engagement can also be understood through interest development theory. Hidi and Renninger (2006) suggest that situational interest can stimulate attention and encourage deeper involvement with learning materials. Consistent with this view, students who reported stronger interest, emotional response, and audiovisual experience also reported higher engagement. Taken together, these findings suggest that animation-based learning is most effective when perceptual, emotional, and cognitive aspects of learning are aligned.

### 5.2 Dual pathways from learning processes to behavioural intention

After establishing that learning experience predicted engagement and perceived comprehension, the study further examined whether these learning processes were associated with behavioural intention. The results showed that engagement and perceived comprehension independently mediated the relationship between learning experience and behavioural intention toward industrial heritage protection. The indirect effect through engagement (*β* = 0.279) and the indirect effect through comprehension (*β* = 0.293) were similar in magnitude, indicating that both pathways contributed to intention formation.

The engagement pathway shows how affective involvement may connect learning experience with behavioural intention. When students are more engaged during learning, they are more likely to develop a personal connection with the learning topic. This interpretation is consistent with educational psychology research showing that emotional involvement and motivational investment can shape attitudes and future behavioural tendencies (Pekrun, 2006; Sinatra et al., 2015). In the present study, emotional response was part of the broader learning experience construct, which helps explain why engagement became one route through which learning experience influenced behavioural intention.

The comprehension pathway provides a complementary explanation. Behavioural intention is not formed only through emotional involvement; it also depends on whether learners understand the meaning and value of the learning content. Previous research in environmental and heritage education has suggested that knowledge, awareness, and meaningful learning experiences are important foundations for conservation-related attitudes and intentions (Ardoin et al., 2020; Ballantyne et al., 2011; Chawla, 2020; Smith, 2006). In this study, students who reported stronger perceived comprehension of industrial heritage content also reported stronger behavioural intention toward heritage protection.

These two pathways therefore point to a broader psychological mechanism. Engagement represents the affective route from learning experience to behavioural intention, whereas perceived comprehension represents the cognitive route. The comparable size of the two indirect effects suggests that neither route should be treated as secondary. Rather, animation-based learning appears to support behavioural intention when it promotes both emotional involvement and perceived comprehension.

### 5.3 Theoretical and practical implications

The findings have several implications for multimedia learning and educational psychology. Theoretically, studying contributes in three ways. First, the quasi-experimental findings provide further evidence that animation-based learning may support stronger educational outcomes than conventional instruction in the context of industrial heritage education. Second, the findings highlight the importance of learners’ subjective learning experiences in shaping both engagement and perceived comprehension during animation-based learning. Third, the results suggest that engagement and perceived comprehension represent complementary pathways through which learning experience is associated with behavioural intention. By integrating learning experience, engagement, perceived comprehension, and behavioural intention within a single framework, the study provides a more comprehensive perspective on how animation-based learning may relate to both learning processes and broader educational outcomes.

The present study also contributes to ongoing discussions of affective-cognitive processes in multimedia learning. Existing models, including the Cognitive-Affective Theory of Learning with Media, have primarily examined how effective and motivational processes support cognitive engagement and immediate learning outcomes (Moreno, 2006). The present results suggest that these processes may also be relevant when educational goals extend beyond knowledge acquisition to include value-based behavioural intentions. This perspective is particularly relevant in industrial heritage education, where learning aims not only to develop children’s understanding of historical and technological knowledge but also to foster appreciation of heritage values and intentions to protect industrial heritage (Smith, 2006; Kisler, 2026). The comparable indirect effects observed for engagement and perceived comprehension suggest that these two processes may provide complementary pathways linking learning experience with behavioural intention. Rather than proposing a new mediation mechanism, the study suggests that an existing effective-cognitive perspective may also help explain how learners’ subjective learning experiences relate to behavioural intention in the context of industrial heritage education. As the study focuses on learners’ perceived comprehension, the observed relationships are best interpreted as reflecting learners’ subjective understanding of the learning experience.

Taken together, these results are consistent with an effective-cognitive perspective on multimedia learning. The significant indirect effects through engagement and perceived comprehension indicate that learning experience may be associated with behavioural intention through both emotional processes and learners’ subjective understanding of the learning content. This finding suggests that the educational value of animation lies not only in supporting learners’ perceived comprehension but also in promoting engagement and meaningful learner involvement throughout the learning process.

From a practical perspective, the findings suggest that educational animation design should balance conceptual clarity with emotional engagement. This recommendation is consistent with multimedia learning research emphasising effective cognitive processing (Mayer, 2020), educational psychology research highlighting the role of emotion in learning (Pekrun, 2006), and recent evidence from technology-enhanced learning showing that well-designed digital learning environments can improve educational effectiveness when instructional approaches are aligned with learners’ needs and instructional objectives (Hessen et al., 2026). If information is communicated clearly but the animation fails to attract attention or encourage emotional involvement, its influence on behavioural intention may be more limited. High-quality audiovisual design, coherent storytelling, and conceptually clear content should therefore be considered complementary rather than independent design features. This also highlights the importance of educators’ capacity to design and implement technology-supported learning effectively (Anggraini et al., 2024).

These implications may be particularly relevant to industrial heritage education. Industrial heritage often involves historical development, technological systems, and environmental transformation processes that can be difficult for young learners to understand through conventional instruction alone. Animation-based storytelling offers one approach to organising such complex content into forms that are more accessible to children. More broadly, the learning experience-engagement-perceived comprehension-behavioural intention framework may provide a useful perspective for future research examining educational contexts in which both knowledge acquisition and attitude development are considered important educational outcomes.

### 5.4 Limitations and future research

Several limitations should be acknowledged. First, the study was conducted with primary school students in a specific regional context in China. Although the sample covered Grades 1 to 6, the findings may not fully generalise to other age groups, cultural contexts, or educational settings. Future research could therefore test whether the proposed model operates similarly among secondary school students, university students, adult learners, or learners from different national and cultural backgrounds.

Second, the study relied on self-report measures. This is appropriate for assessing students’ perceived learning experience, engagement, comprehension, and behavioural intention. Still, it also means that the findings may be influenced by social desirability bias or by students’ perceived rather than actual understanding. Future studies could address this limitation by incorporating objective knowledge tests, behavioural observations, eye-tracking measures, or physiological indicators of engagement. Such data would provide a more comprehensive understanding of how animation-based learning affects both perceived and observable learning outcomes.

Third, the study examined a single animation based on the Dagushan industrial heritage site. As a result, the observed effects cannot be fully separated from the specific content, narrative structure, duration, visual style, or instructional design of this animation. Future research could compare multiple animations with different design features to examine how narrative structure, audiovisual design, interactivity, and content complexity influence learning processes and behavioural intention. This would help clarify which design features are most important for supporting affective and cognitive pathways in animation-based learning.

Despite these limitations, the present study contributes to multimedia learning research by showing that learning experience influences behavioural intention through both engagement and comprehension. The findings also suggest that animation-based learning can support young learners’ understanding of complex content while engaging both affective and cognitive learning processes.

## 6 Conclusion

This study examined how animation-based learning influences primary school students’ behavioural intention toward industrial heritage protection by proposing and testing a perception–process–outcome model in which learning experience influences behavioural intention through engagement and perceived comprehension. Using a quasi-experimental design involving 452 primary school students and structural equation modeling with Bootstrap mediation analysis, the study investigated both the effectiveness of the animation intervention and the psychological mechanisms underlying its effects.

Three main findings emerged. First, compared with conventional instruction, the animation-based intervention produced significantly greater improvements in students’ interest, perceived comprehension, and behavioural intention toward industrial heritage protection. Second, the structural model supported all hypothesised relationships. Learning experience positively influenced both engagement and perceived comprehension, while engagement and perceived comprehension were both positively associated with behavioural intention. Third, Bootstrap mediation analysis confirmed that learning experience influenced behavioural intention through both effective and cognitive pathways.

The findings contribute to multimedia learning and educational psychology research in several ways. First, they broaden understanding of animation-based learning by demonstrating how learning experience, engagement, perceived comprehension, and behavioural intention are associated within a single framework in an industrial heritage education context. Second, they provide empirical evidence that affective engagement and perceived comprehension may represent complementary pathways through which learning experience is associated with behavioural intention. Third, they highlight the importance of learners’ subjective experiences, including interest, emotional response, and audiovisual experience, in shaping both learning processes and broader educational outcomes. Together, these findings suggest that the study’s contribution lies not in proposing a new multimedia learning mechanism but in demonstrating how an established affective-cognitive perspective may also help explain behavioural intention within an industrial heritage education context.

From a practical perspective, the findings suggest that educational animations may be particularly effective when they combine coherent storytelling, meaningful emotional engagement, and conceptually clear content. Narrative-based approaches may be especially valuable when complex industrial processes, historical developments, and environmental transformations are communicated through human-centred stories that are meaningful to young learners. Such design characteristics appear to support both engagement and perceived comprehension, which in turn contribute to behavioural intention. Although the present study was situated within the context of industrial heritage education, the proposed framework may also be relevant to other domains involving complex knowledge communication, public awareness, and educational behaviour change.

Overall, the study demonstrates that animation-based learning can support not only learners’ perceived comprehension but also positive behavioural intentions through complementary affective and cognitive processes, highlighting its potential as an educational tool for communicating complex industrial heritage content to young learners.

## Data Availability

The original contributions presented in the study are included in the article/Supplementary material, further inquiries can be directed to the corresponding author.

## References

[ref1] Abas R. Mohamad Asri S. F. Saat A. Mohd Nor N. H. Hod R. Mohd Nizam D. N. . (2022). A review of multimedia usage in embryology education. Asia-Pac. J. Inf. Technol. Multimedia 11, 1–11. doi: 10.17576/apjitm-2022-1101-01

[ref2] Anggraeni A. D. Pentury J. Tanamal A. (2019). Blending humour and animation in english learning for students of english education programs in universities. Int. J. Innov. Creat. Change 5, 540–553.

[ref3] Anggraini T. Ahmad M. Hanafi I. (2024). Digital literacy and teaching experience as predictors of pedagogical competence in the digital era. Tarbawi: Jurnal Keilmuan Manajemen Pendidikan 10, 295–306. doi: 10.32678/tarbawi.v10i02.10795

[ref4] Ardoin N. M. Bowers A. W. Gaillard E. (2020). Environmental education outcomes for conservation: a systematic review. Biol. Conserv. 241:108224. doi: 10.1016/j.biocon.2019.108224

[ref5] Ashfaq H. Ansari M. N. (2020). Experiences of online reading. Libr. Philos. Pract. 4131, 1–15.

[ref6] Bakan U. Han T. Bakan U. (2025). Exploring the impact of the uncanny valley effect on teenagers’ attitudes toward virtual characters and EFL reading comprehension skills. Intell. Serv. Robot. 18, 233–246. doi: 10.1007/s11370-024-00575-w

[ref7] Ballantyne R. Packer J. Falk J. (2011). Visitors’ learning for environmental sustainability: testing short-and long-term impacts of wildlife tourism experiences using structural equation modelling. Tour. Manag. 32, 1243–1252. doi: 10.1016/j.tourman.2010.11.003

[ref8] Bian X. Brown A. Marques B. (2025a). Examining a primary education approach using digital storytelling: Chinese industrial heritage as a vehicle to support learning. Heritage 8:477. doi: 10.3390/heritage8110477

[ref9] Bian X. Brown A. Marques B. (2025b). Heritage appreciation and awareness: a child educational approach exploiting animated video. Int. J. Art Des. Educ. 44, 286–304. doi: 10.1111/jade.12563

[ref10] Bian X. Brown A. Marques B. (2026a). Balancing ecological restoration and industrial landscape heritage values through a digital narrative approach: a case study of the Dagushan Iron mine, China. Land 15:155. doi: 10.3390/land15010155

[ref11] Bian X. Brown A. Marques B. (2026b). Digital storytelling for primary heritage learning: early sustainability relevant meaning-making in an industrial heritage case. Sustainability 18:2319. doi: 10.3390/su18052319

[ref12] Chawla L. (2020). Childhood nature connection and constructive hope: a review of research on connecting with nature and coping with environmental loss. People Nat. 2, 619–642. doi: 10.1002/pan3.10128

[ref13] Chen C.-C. Chai M.-H. Lin P.-H. (2025). Exploring the impact of interactive multimedia e-books on the effectiveness of environmental learning, pro-environmental attitudes, and behavioural intentions among primary school students. J. Comput. Assist. Learn. 41:e70087. doi: 10.1111/jcal.70087

[ref14] Elbeshbeishy R. Salama R. Goud B. K. M. Babiker R. Jhancy M. Hamed N. . (2025). Unveiling the perceptions of medical and allied health students towards cadaveric dissection and virtual resources in anatomy education: a cross sectional study. BMC Med. Educ. 25:829. doi: 10.1186/s12909-025-07432-z, 40462035 PMC12135608

[ref15] Fredricks J. A. Blumenfeld P. C. Paris A. H. (2004). School engagement: potential of the concept, state of the evidence. Rev. Educ. Res. 74, 59–109. doi: 10.3102/00346543074001059

[ref16] Guo M. (2025). Bichronous modes in heritage education for enhancing motivation and learning outcomes via the ARCS model. NPJ Herit. Sci. 13:268. doi: 10.1038/s40494-025-01858-w

[ref17] Hessen B. T. E. Hewary O. A. Fileen N. G. (2026). When artificial intelligence meets blended learning: a quasi-experimental study of physics achievement at the University of Khartoum, Sudan. Tarbawi: Jurnal Keilmuan Manajemen Pendidikan 12, 29–40. doi: 10.32678/tarbawi.v12i01.11360

[ref18] Hidi S. Renninger K. A. (2006). The four-phase model of interest development. Educ. Psychol. 41, 111–127. doi: 10.1207/s15326985ep4102_4

[ref19] Hoyt G. Bakshi C. S. Basu P. (2025). Integration of an audiovisual learning resource in a podiatric medical infectious disease course: multiple cohort pilot study. JMIR Med. Educ. 11:e55206. doi: 10.2196/55206, 39935004 PMC11835597

[ref20] Kim S.-i. Yoon M. Whang S. M. Tversky B. Morrison J. B. (2007). The effect of animation on comprehension and interest. J. Comput. Assist. Learn. 23, 260–270. doi: 10.1111/j.1365-2729.2006.00219.x

[ref21] Kisler R. (2026). Mapping heritage education: historical knowledge and moral values. Stud. Philos. Educ. 45, 439–458. doi: 10.1007/s11217-025-10037-0

[ref9001] Kock N. (2015). Common method bias in PLS-SEM: a full collinearity assessment approach. Int. J. e-Collab. 11, 1–10. doi: 10.4018/IJeC.2015100101

[ref22] Lee R. K. Y. Ng B. Y. N. Chen D. M. H. (2019). Using metro lines for integration of nucleotide metabolic pathways. Biochem. Mol. Biol. Educ. 47, 532–537. doi: 10.1002/bmb.21256, 31125165

[ref23] Leshko D. Yablon L. Hasiuk I. (2025). The principle of historicism and ways of its implementation in a physics course. J. Vasyl Stefanyk Precarpathian National University 12, 96–104. doi: 10.15330/jpnu.12.1.96-104

[ref24] Liu H. Yao C. Zhang Y. Ban X. (2024). GestureTeach: a gesture guided online teaching interactive model. Comput. Anim. Virtual Worlds 35:e2218. doi: 10.1002/cav.2218

[ref25] Lowe R. K. (1999). Extracting information from an animation during complex visual learning. Eur. J. Psychol. Educ. 14, 225–244. doi: 10.1007/BF03172967

[ref26] Lu R.-S. Lin H.-C. K. Yang Y.-C. Chen Y.-P. (2024). Integrating urban mining concepts through AI-generated storytelling and visuals: advancing sustainability education in early childhood. Sustainability 16:11304. doi: 10.3390/su162411304

[ref27] Luck S. J. Hollingworth A. R. (2008). Visual Memory. Oxford: Oxford University Press. doi: 10.1093/acprof/9780195305487.001.0001

[ref28] Mahadevaswamy Praveena K. S. Sathyanarayana N. Bhargavi K. Patil C. M. (2025). Tool based teaching for enhancing the learner outcome in digital communication and wireless sensor network course: a case study. J. Eng. Educ. Transform. 38, 574–583. doi: 10.16920/jeet/2025/v38is2/25071

[ref29] Marzouk M. A. Ibrahim S. S. (2026). AI-driven eye-tracking and emotion recognition to improve attention across storytelling modalities in children with autism spectrum disorder. Int. J. Hum.-Comput. Interact., 1–23. doi: 10.1080/10447318.2026.2633206, 37339054

[ref30] Mayer R. E. (2020). Multimedia Learning. 3rd ed. Cambridge: Cambridge University Press.

[ref31] Moreno R. (2006). Learning in high-tech and multimedia environments. Curr. Dir. Psychol. Sci. 15, 63–67. doi: 10.1111/j.0963-7214.2006.00408.x

[ref32] Pekrun R. (2006). The control-value theory of achievement emotions: assumptions, corollaries, and implications for educational research and practice. Educ. Psychol. Rev. 18, 315–341. doi: 10.1007/s10648-006-9029-9

[ref33] Ploetzner R. Lowe R. (2012). A systematic characterisation of expository animations. Comput. Hum. Behav. 28, 781–794. doi: 10.1016/j.chb.2011.12.001

[ref34] Podsakoff P. M. MacKenzie S. B. Lee J. Y. Podsakoff N. P. (2003). Common method biases in behavioral research: a critical review of the literature and recommended remedies. J. Appl. Psychol. 88, 879–903. doi: 10.1037/0021-9010.88.5.879, 14516251

[ref35] Roohani A. Jafarpour A. Zarei S. (2015). Effects of visualisation and advance organisers in reading multimedia-based texts. 3L Southeast Asian J. Eng. Lang. Stud. 21, 47–62. doi: 10.17576/3L-2015-2102-04

[ref36] Ross K. M. Pye R. E. Randell J. (2016). Reading touch screen storybooks with mothers negatively affects 7-year-old readers’ comprehension but enriches emotional engagement. Front. Psychol. 7:1728. doi: 10.3389/fpsyg.2016.01728, 27899903 PMC5110538

[ref37] Sandaran S. C. Kia L. C. (2013). The use of digital stories for listening comprehension among primary Chinese medium school pupils: some preliminary findings. J. Teknol. 65, 125–131.

[ref38] Schnotz W. Rasch T. (2005). Enabling, facilitating, and inhibiting effects of animations in multimedia learning: why reduction of cognitive load can have negative results on learning. Educ. Technol. Res. Dev. 53, 47–58. doi: 10.1007/BF02504797

[ref39] Schurgin M. W. (2018). Visual memory, the long and the short of it: a review of visual working memory and long-term memory. Atten. Percept. Psychophys. 80, 1035–1056. doi: 10.3758/s13414-018-1522-y, 29687357

[ref40] Sinatra G. M. Heddy B. C. Lombardi D. (2015). The challenges of defining and measuring student engagement in science. Educ. Psychol. 50, 1–13. doi: 10.1080/00461520.2014.1002924

[ref41] Smith L. (2006). Uses of Heritage. Abingdon: Routledge.

[ref42] Subramanian R. R. Inukurthi B. (2026). Manime: a code-driven visual teaching method for deep learning education. J. Eng. Educ. Transform. 39, 381–388. doi: 10.16920/jeet/2026/v39is2/26046

[ref43] Sweller J. (2011). “Cognitive load theory,” in Psychology of Learning and Motivation: Cognition in Education, eds. Mestre J. P. Ross B. H. (San Diego: Academic Press), 55, 37–76. doi: 10.1016/B978-0-12-387691-1.00002-8

[ref44] Thatcher J. D. (2006). Computer animation and improved student comprehension of basic science concepts. J. Osteopath. Med. 106, 9–14. doi: 10.1201/9781420006155.ch1, 16428683

[ref45] Tsehay Mengistie S. (2025). Effect of simulation and animation-integrated technology instruction on pre-service geography teacher trainees' conceptual understanding and retention in learning the earth's rotation and revolution. Int. Res. Geogr. Environ. Educ. 34, 1–22. doi: 10.1080/10382046.2025.2531014, 37339054

[ref46] Turkay S. (2022). Comparison of dynamic visuals to other presentation formats when learning social science topics in an online setting. Australas. J. Educ. Technol. 38, 12–26. doi: 10.14742/ajet.7639

[ref47] Um E. Plass J. L. Hayward E. O. Homer B. D. (2012). Emotional design in multimedia learning. J. Educ. Psychol. 104, 485–498. doi: 10.1037/a0026609

[ref48] Wagino W. Abidin Z. Ekasari D. Pujiastuti A. Sukartiningsih W. (2025). Language skills of deaf students using the computer assisted instruction (CAI) application program model. Int. J. Lang. Educ. 9, 531–548. doi: 10.26858/ijole.v1i1.77395

[ref49] Yang J. Sun Y. Wang L. Yang S. Zhang J. (2026). Difficulty-based three-dimensional animation in oral histology and embryology education: an explorative study. Eur. J. Dent. Educ. 30, 599–608. doi: 10.1111/eje.70006, 40542511

