# Supplementary material for: From learning experience to behavioural intention: examining the effects of animation-based industrial heritage learning among primary school students
Source: Front Psychol. 2026 Aug 24;17:1907096. doi: 10.3389/fpsyg.2026.1907096 (PMC13547045; doi:10.3389/fpsyg.2026.1907096)
Supplement: Supplementary file 1 [file Table_1.DOCX]

**Supplementary Table S1.** Standardised factor loadings, composite reliability (CR), and average variance extracted (AVE) for the measurement model

Table S1. Standardised factor loadings, composite reliability (CR), and average variance extracted (AVE) for the measurement model

| **Dimension** | **Item** | **Standardized Loading** | **CR** | **AVE** |
| --- | --- | --- | --- | --- |
| Interest | INT1 | 0.783 | 0.830 | 0.620 |
|  | INT2 | 0.793 |  |  |
|  | INT3 | 0.786 |  |  |
| Emotional Response | ER1 | 0.802 | 0.831 | 0.621 |
|  | ER2 | 0.749 |  |  |
|  | ER3 | 0.813 |  |  |
| Visual & Auditory Experience | VAE1 | 0.830 | 0.837 | 0.632 |
|  | VAE2 | 0.747 |  |  |
|  | VAE3 | 0.806 |  |  |
| Engagement | ENG1 | 0.734 | 0.826 | 0.614 |
|  | ENG2 | 0.803 |  |  |
|  | ENG3 | 0.811 |  |  |
| Comprehension | COM1 | 0.762 | 0.828 | 0.617 |
|  | COM2 | 0.804 |  |  |
|  | COM3 | 0.789 |  |  |
| Behavioural Intention | BI1 | 0.804 | 0.847 | 0.649 |
|  | BI2 | 0.798 |  |  |
|  | BI3 | 0.814 |  |  |

Note. All loadings significant at p < .001. CR = (Σλ)²/[(Σλ)²+Σ(1−λ²)]; AVE = Σλ²/n. All constructs exceed the CR ≥ .70 and AVE ≥ .50 thresholds (Fornell & Larcker, 1981).

**Supplementary Table S2.** Heterotrait–monotrait ratio (HTMT) matrix

Table S2. Heterotrait–monotrait ratio (HTMT) matrix

| **Dimension** | **Interest** | **Emot. Response** | **Vis. &**  **Aud. Exp.** | **Engagement** | **Comprehension** | **Beh. Intention** |
| --- | --- | --- | --- | --- | --- | --- |
| Interest | — |  |  |  |  |  |
| Emotional Response | 0.244 | — |  |  |  |  |
| Visual & Auditory Exp. | 0.331 | 0.258 | — |  |  |  |
| Engagement | 0.376 | 0.353 | 0.378 | — |  |  |
| Comprehension | 0.416 | 0.420 | 0.433 | 0.267 | — |  |
| Behavioural Intention | 0.376 | 0.463 | 0.410 | 0.695 | 0.661 | — |

Note. All HTMT values < .85, supporting discriminant validity between all construct pairs (Henseler et al., 2015).

**Supplementary Table S3.** Modification indices (MI) for the measurement model (top 20 of 243 candidate parameters examined)

Table S3. Modification indices (MI) for the measurement model (top 20 of 243 candidate parameters examined)

| **Rank** | **Type** | **Parameter** | **MI (Δχ², df=1)** | **Std. EPC** |
| --- | --- | --- | --- | --- |
| 1 | Residual covariance | VAE3 ↔ ENG2 | 6.858 | 0.085 |
| 2 | Residual covariance | INT1 ↔ BI3 | 6.423 | −0.082 |
| 3 | Cross-loading | ER2 → Comprehension | 5.470 | 0.145 |
| 4 | Residual covariance | VAE2 ↔ BI1 | 4.998 | 0.074 |
| 5 | Residual covariance | VAE1 ↔ BI1 | 4.895 | −0.068 |
| 6 | Residual covariance | INT3 ↔ ENG2 | 4.320 | −0.070 |
| 7 | Residual covariance | VAE1 ↔ ENG2 | 4.154 | −0.065 |
| 8 | Residual covariance | BI1 ↔ BI3 | 4.133 | −0.086 |
| 9 | Residual covariance | ENG1 ↔ ENG3 | 4.043 | −0.104 |
| 10 | Cross-loading | INT1 → Behavioural Intention | 3.977 | −0.129 |
| 11 | Residual covariance | INT1 ↔ ER1 | 3.128 | 0.060 |
| 12 | Residual covariance | VAE1 ↔ BI3 | 3.116 | 0.054 |
| 13 | Cross-loading | ENG3 → Interest | 3.116 | 0.106 |
| 14 | Residual covariance | INT2 ↔ ER1 | 3.035 | −0.059 |
| 15 | Residual covariance | ER2 ↔ VAE1 | 3.001 | 0.059 |
| 16 | Cross-loading | BI2 → Visual & Auditory Exp. | 2.959 | −0.105 |
| 17 | Residual covariance | ER1 ↔ ENG2 | 2.882 | −0.056 |
| 18 | Cross-loading | ER3 → Visual & Auditory Exp. | 2.872 | −0.099 |
| 19 | Cross-loading | ER3 → Comprehension | 2.825 | −0.114 |
| 20 | Residual covariance | INT3 ↔ BI2 | 2.711 | 0.054 |

Note. MI computed as Δχ² (1 df) from freeing each parameter and refitting. All 243 candidates (153 residual covariances + 90 cross-loadings) examined; maximum MI = 6.858, well below the conventional threshold of 10 — no respecification needed.

**Supplementary Table S4.** Assessment of Potential Common Method Bias

Table S4. Assessment of Potential Common Method Bias

| Test | Index | Criterion | Observed Result | Conclusion |
| --- | --- | --- | --- | --- |
| Harman's single-factor test | Variance explained by first unrotated factor | <50% | 34.08% (five factors with eigenvalues >1; cumulative variance=71.80%) | No evidence of substantial common method bias |
| Confirmatory single-factor test (CFA) | Six-factor vs one-factor model | One-factor model should fit substantially worse | Six-factor: χ²=108.60, df=120, p=.763, CFI=1.01, RMSEA<.001; One-factor: χ²=972.99, df=135, p<.001, CFI=.55, RMSEA=.17; Δχ²(15)=864.39, p<.001 | Results indicate that a single common factor does not adequately explain the observed covariance structure. |

Note. Harman's single-factor test and the comparison between the hypothesised six-factor measurement model and an alternative single-factor model consistently indicated that common method bias was unlikely to have materially influenced the findings.

**Supplementary Table S5.** Questionnaire items used in the study

All experiential variables were measured using a five-point Likert scale ranging from 1 = Strongly disagree to 5 = Strongly agree. The questionnaire comprised six constructs and eighteen items.

Table S5. Questionnaire items used in the study

| **Construct** | **Code** | **Questionnaire item** |
| --- | --- | --- |
| Interest | INT1 | I found the animation interesting. |
|  | INT2 | I would recommend this animation to my friends. |
|  | INT3 | The animation captured my attention. |
| Emotional Response | ER1 | The animation moved or inspired me emotionally. |
|  | ER2 | The story in the animation resonated with me emotionally. |
|  | ER3 | I felt emotionally connected with the characters or events in the animation. |
| Audiovisual Experience | AE1 | The animation visuals were vivid and engaging. |
|  | AE2 | The background music and sound effects enhanced my viewing experience. |
|  | AE3 | The visual and audio presentation helped me understand the content better. |
| Engagement | ENG1 | I watched the animation attentively. |
|  | ENG2 | The animation maintained my interest throughout. |
|  | ENG3 | After watching the animation, I wanted to learn more about the topic. |
| Perceived Comprehension | COM1 | I can explain the main story presented in the animation about the Dagushan Iron Mine. |
|  | COM2 | I can describe the basic information about industrial heritage presented in the animation. |
|  | COM3 | After watching the animation, I understood the purpose and historical role of the Dagushan Iron Mine. |
| Protection and Environmental Stewardship Intention (PESI) | PESI1 | The animation helped me better understand industrial heritage. |
|  | PESI2 | I would be willing to participate in activities related to protecting industrial heritage. |
|  | PESI3 | The animation increased my interest in protecting industrial heritage. |

Note. All items were measured using a five-point Likert scale (1 = Strongly disagree to 5 = Strongly agree). Higher scores indicate higher levels of the corresponding construct.
